# Validation of 3 Computer-Aided Facial Phenotyping Tools (DeepGestalt, GestaltMatcher, and D-Score): Comparative Diagnostic Accuracy Study

**DOI:** 10.2196/42904

**Published:** 2024-03-13

**Authors:** Alisa Maria Vittoria Reiter, Jean Tori Pantel, Magdalena Danyel, Denise Horn, Claus-Eric Ott, Martin Atta Mensah

**Affiliations:** 1 Institute of Medical Genetics and Human Genetics Charité - Universitätsmedizin Berlin, corporate member of Freie Universität Berlin and Humboldt-Universität zu Berlin Berlin Germany; 2 Institute for Digitalization and General Medicine University Hospital Aachen Aachen Germany; 3 Center for Rare Diseases Aachen ZSEA University Hospital Aachen Aachen Germany; 4 BIH Biomedical Innovation Academy Clinician Scientist Program Berlin Institute of Health at Charité–Universitätsmedizin Berlin Berlin Germany; 5 Berlin Center for Rare Diseases Charité - Universitätsmedizin Berlin corporate member of Freie Universität Berlin and Humboldt-Universität zu Berlin Berlin Germany; 6 BIH Biomedical Innovation Academy Digital Clinician Scientist Program Berlin Institute of Health at Charité–Universitätsmedizin Berlin Berlin Germany

**Keywords:** facial phenotyping, DeepGestalt, facial recognition, Face2Gene, medical genetics, diagnostic accuracy, genetic syndrome, machine learning, GestaltMatcher, D-Score, genetics

## Abstract

**Background:**

While characteristic facial features provide important clues for finding the correct diagnosis in genetic syndromes, valid assessment can be challenging. The next-generation phenotyping algorithm DeepGestalt analyzes patient images and provides syndrome suggestions. GestaltMatcher matches patient images with similar facial features. The new D-Score provides a score for the degree of facial dysmorphism.

**Objective:**

We aimed to test state-of-the-art facial phenotyping tools by benchmarking GestaltMatcher and D-Score and comparing them to DeepGestalt.

**Methods:**

Using a retrospective sample of 4796 images of patients with 486 different genetic syndromes (London Medical Database, GestaltMatcher Database, and literature images) and 323 inconspicuous control images, we determined the clinical use of D-Score, GestaltMatcher, and DeepGestalt, evaluating sensitivity; specificity; accuracy; the number of supported diagnoses; and potential biases such as age, sex, and ethnicity.

**Results:**

DeepGestalt suggested 340 distinct syndromes and GestaltMatcher suggested 1128 syndromes. The top-30 sensitivity was higher for DeepGestalt (88%, SD 18%) than for GestaltMatcher (76%, SD 26%). DeepGestalt generally assigned lower scores but provided higher scores for patient images than for inconspicuous control images, thus allowing the 2 cohorts to be separated with an area under the receiver operating characteristic curve (AUROC) of 0.73. GestaltMatcher could not separate the 2 classes (AUROC 0.55). Trained for this purpose, D-Score achieved the highest discriminatory power (AUROC 0.86). D-Score’s levels increased with the age of the depicted individuals. Male individuals yielded higher D-scores than female individuals. Ethnicity did not appear to influence D-scores.

**Conclusions:**

If used with caution, algorithms such as D-score could help clinicians with constrained resources or limited experience in syndromology to decide whether a patient needs further genetic evaluation. Algorithms such as DeepGestalt could support diagnosing rather common genetic syndromes with facial abnormalities, whereas algorithms such as GestaltMatcher could suggest rare diagnoses that are unknown to the clinician in patients with a characteristic, dysmorphic face.

## Introduction

### Background

Particularly in genetic syndromology, specific external anatomical features of patients are used to evaluate differential diagnoses [1]. Many inheritable syndromes are associated with specific patterns of dysmorphic signs, especially of the face. However, the clinical genetic description of a patient’s face is complicated, and the perception and naming of abnormalities depend on the individual examiner [2]. Establishing a clinical genetic differential diagnosis is also complicated by the plethora of rare genetic syndromes [3].

Computer vision and machine learning offer scalable technologies that allow rapid and standardized recognition of relevant facial anatomical parameters and automatic assignment to a list of possible diagnoses. Several tools have been developed (eg, [4-13]) that perform computer-aided facial phenotyping using common patient photographs or even 3D patient images, often referred to as next-generation phenotyping (NGP). Ideally, NGP tools succeed in three tasks: (1) discerning patients from unaffected controls, (2) recognizing as many syndromes as possible, and (3) assigning the correct diagnosis.

One provider of such NGP tools is Face2Gene (FDNA Inc), which offers a range of applications based on the DeepGestalt [10] neural network. Initial studies have evaluated the ability of this network to correctly detect a syndrome in a patient image, suggesting good sensitivity (eg, [14-21]). It has been shown that such tools can also be trained for yet unknown syndromes by demonstrating that a new syndrome has a specific gestalt that can be distinguished from other syndromes (eg, [22-29]). DeepGestalt can only detect a specific syndrome if a minimum number of images of that syndrome is included in the training set. However, the GestaltMatcher tool—a further development of DeepGestalt—solves this problem by matching patient images against each other so that extremely rare or unknown syndromes can be detected. The last layer of the neural network is used to create a multidimensional clinical face phenotype space in which the spatial proximity reflects the similarity between 2 patient faces [30]. Among other studies, these findings suggest that facial NGP could advance the diagnostic process in medical genetics as well as support and accelerate the detection of novel syndromes. More recently, computer-aided facial phenotyping has been discussed for screening in telehealth applications, especially in resource-limited settings [13]. While DeepGestalt, in principle, is able to distinguish patients who are dysmorphic from unaffected control individuals [20,22], it by design has a limited ability to produce markedly different scores on photographs of affected and unaffected individuals [20,21]. The latest addition to the Face2Gene application suite called D-Score*,* however*,* aims to provide a discriminatory score to identify patients with dysmorphic facial features.

### Objective

The recently described GestaltMatcher has not yet been benchmarked in individuals without a genetic syndrome and D-Score has not been benchmarked at all. Therefore, we evaluated the diagnostic accuracy of D-Score in individuals of different sexes, ages, and ethnic backgrounds and compared the results with those of DeepGestalt and GestaltMatcher.

## Methods

### Deep Learning Models

#### DeepGestalt

DeepGestalt is a deep convolutional neural network, trained on portrait images of individuals with genetic syndromes as described in detail previously [10]. The classes of the network are defined by sets of images of specific syndromes. When DeepGestalt analyzes the facial image of the query, it calculates and assigns a score for each of these classes, which corresponds to the degree of similarity of the query image to that class. The tool returns a list of syndrome suggestions ordered by the so-called DeepGestalt score. Here, we used DeepGestalt version 21.5.0 to obtain the top-30 syndrome matches and DeepGestalt scores for each image of the included test sets.

#### GestaltMatcher

GestaltMatcher is a previously described [30] encoder for portrait photos based on a deep convolutional neural network that creates a clinical face phenotype space [7] to match patient portrait images by the similarity of facial features. This tool can be used either to determine the similarity of different query images to each other or to individually embedded images, or to get syndrome suggestions for a single query image. In this study, we only tested GestaltMatcher’s latter ability. For the analysis of the test sets with GestaltMatcher (version 0.8.0), the maximum possible number (n=100) of suggested syndromes in the web interface was chosen to count the number of syndromes known to this system, including syndromes supported by DeepGestalt as well as ultrarare syndromes not supported by DeepGestalt. When comparing the rank-dependent sensitivity of GestaltMatcher and DeepGestalt, however, only the first 30 suggestions of GestaltMatcher were used, since DeepGestalt only provides 30 suggestions.

#### D-Score

D-score is a support vector machine running on the output of a deep convolutional neural network for the syndrome classification of patient images. As a binary classifier, D-Score returns a single value per image, aiming to capture the degree of facial dysmorphism. The concept has been described previously [20]. However, D-Score was trained and validated on a larger data set and its architecture is more advanced and more complex than the model described by Pantel et al [20]. The test sets were analyzed by the beta version of D-Score (version 0.1 beta) accessed via an applied programming interface.

### Test Sets

#### Overview

**Figure 1:**
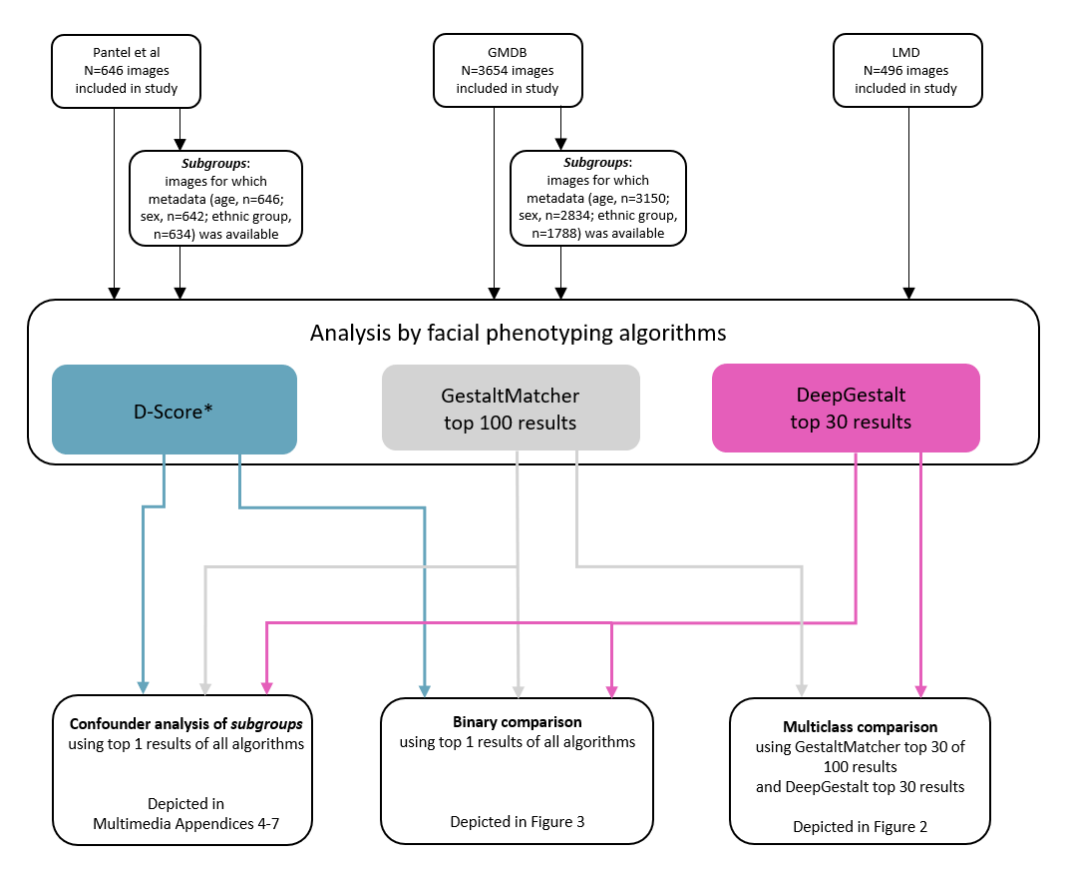
Workflow of analyses. GMDB: GestaltMatcher Database; LMD: London Medical Database. *Binary classifier uses only 1 score.

In this study, we analyzed images of 3 data sets (London Medical Database [LMD], GestaltMatcher Database [GMDB], and data previously described in Pantel et al [20]; Figure 1 [20] and Table 1) as a retrospective sample. We aimed to analyze as many cases as possible using only 1 frontal image per case and restricting the test set to those cases that are not included in the training sets of DeepGestalt, GestaltMatcher, or D-Score. For syndromic cases, the diagnosis was established either clinically or molecularly. No other information was entered into the facial analysis applications besides the frontal images.

**Table 1:** Characteristics of the image data sets.

		Pantel et al [20] syndromic cohort, n	Pantel et al [20] healthy control cohort, n	LMD^a^ test set, n	GMDB^b^ test set, n	Total, N
Included images (total)		323	323	496	3654	4796
D-Score		322	323	496	3654	4795
Failed D-Score		1	0	0	0	1
DeepGestalt		323	323	496	3654	4796
GestaltMatcher		323	321	496	3654	4794
Failed GestaltMatcher		0	2	0	0	2
Number of syndromes		17	N/A^c^	199	346	486
**Sex**						
	Female	159	159	—^d^	1299	1617
	Male	162	162	—	1535	1859
	Unknown	2	2	496	820	1320
**Ethnicity**						
	White	273	273	—	1221	1767
	Other	44	44	—	567	655
	Unknown	6	6	496	1866	2374

^a^LMD: London Medical Database.

^b^GMDB: Gestalt Matcher Database.

^c^N/A: not applicable.

^d^Not available.

#### London Medical Database Set

This test set was built from 496 images of the LMD showing individuals with a genetic syndrome.

#### GestaltMatcher Database Set

The GMDB is a user-curated database of facial images of individuals with genetic syndromes. From this database, 3654 portrait photographs were included in our study that were not part of the training sets.

### Literature Images

This data set was previously used by Pantel et al [20] comprising a total of 646 images, including 323 images of clinically or molecularly diagnosed patients (Pantel syndromic cohort) with 17 genetic syndromes (19 images for each syndrome) and 323 sex-, ethnicity-, and age-matched control images (Pantel healthy control cohort).

### Ethical Considerations

 This study was approved by the ethics committee of the Charité-Universitätsmedizin Berlin (EA2/190/16).

### Statistical Analyses

#### Overview

Statistical analyses were performed using Python (version 3.8; Python Software Foundation). The code and data required to reproduce statistical analyses can be found in Multimedia Appendix 1 (see also the readme.txt for further information).

#### Comparison of DeepGestalt and GestaltMatcher

The algorithms performing a multiclass comparison—that is, DeepGestalt and GestaltMatcher—were compared regarding their numbers of supported syndromes, distributions and heights of yielded scores, sensitivities, and false positive rates. Since DeepGestalt’s output was limited to 30 suggestions, for reason of comparability, a syndrome was also only considered to be supported if it was suggested at least once on rank 30 or better in GestaltMatcher. Top-1, top-10, and top-30 sensitivities, that is, the ability to respectively place the correct diagnosis on the first, within the first 10, or within the first 30 ranks of the results list, were calculated for each supported syndrome represented by at least 5 cases in the testing data sets. Sensitivities were additionally calculated for the images of patients in the literature image set. Mean sensitivities were averaged by the syndrome, not by the number of images, giving each syndrome the same weight. Any syndrome suggestion other than the syndrome featured by the person depicted was considered a false positive. As the tools do not support a class label “inconspicuous face,” any suggestion was defined as a false positive in unaffected controls. Top-1, top-5, top-10, and top-30 false positive rates of each syndrome falsely suggested by a tool were calculated, representing the fraction of cases in which a syndrome was falsely suggested as the top-1 or within the first 5, 10, and 30 suggestions, respectively. False positive rates of patient and control images were tested for a possible correlation using a linear regression model and Pearson correlation coefficient.

#### Validation of the Binary Classification Performance of DeepGestalt, GestaltMatcher, and D-Score

We tested the ability of all 3 models (DeepGestalt, GestaltMatcher, and D-Score) to distinguish portrait images of individuals affected with a genetic syndrome from those of healthy controls. As they return a list of scores, only the highest scores (top-1 scores) assigned to an image by DeepGestalt and GestaltMatcher were compared with the single number output of D-Score. Mann-Whitney *U* tests were used to analyze possible differences in the yielded scores (1-sided for dysmorphic vs nondysmorphic cohorts and 2-sided for any other comparisons). The area under the receiver operating characteristic curve was calculated as a metric of accuracy. Analyses were also performed for clinically relevant patient-specific subgroups. For images for which this information was available, output data were separated by age group, sex, and ethnicity. We used the age groups 0 to 2 years (babies and toddlers), 3 to 10 years (children), 11 to 20 years (teenagers), 21 to 40 years (young adults), 41 to 60 years (middle-aged adults), and older than 60 years (older adults). As the ethnicity of most images was White (defined as being of apparently solely European descent), we built 2 groups of ethnicities: White and other. We did not define a cutoff for syndromic or inconspicuous for any of the tools.

## Results

### Syndrome Classification With DeepGestalt and GestaltMatcher

**Figure 2:**
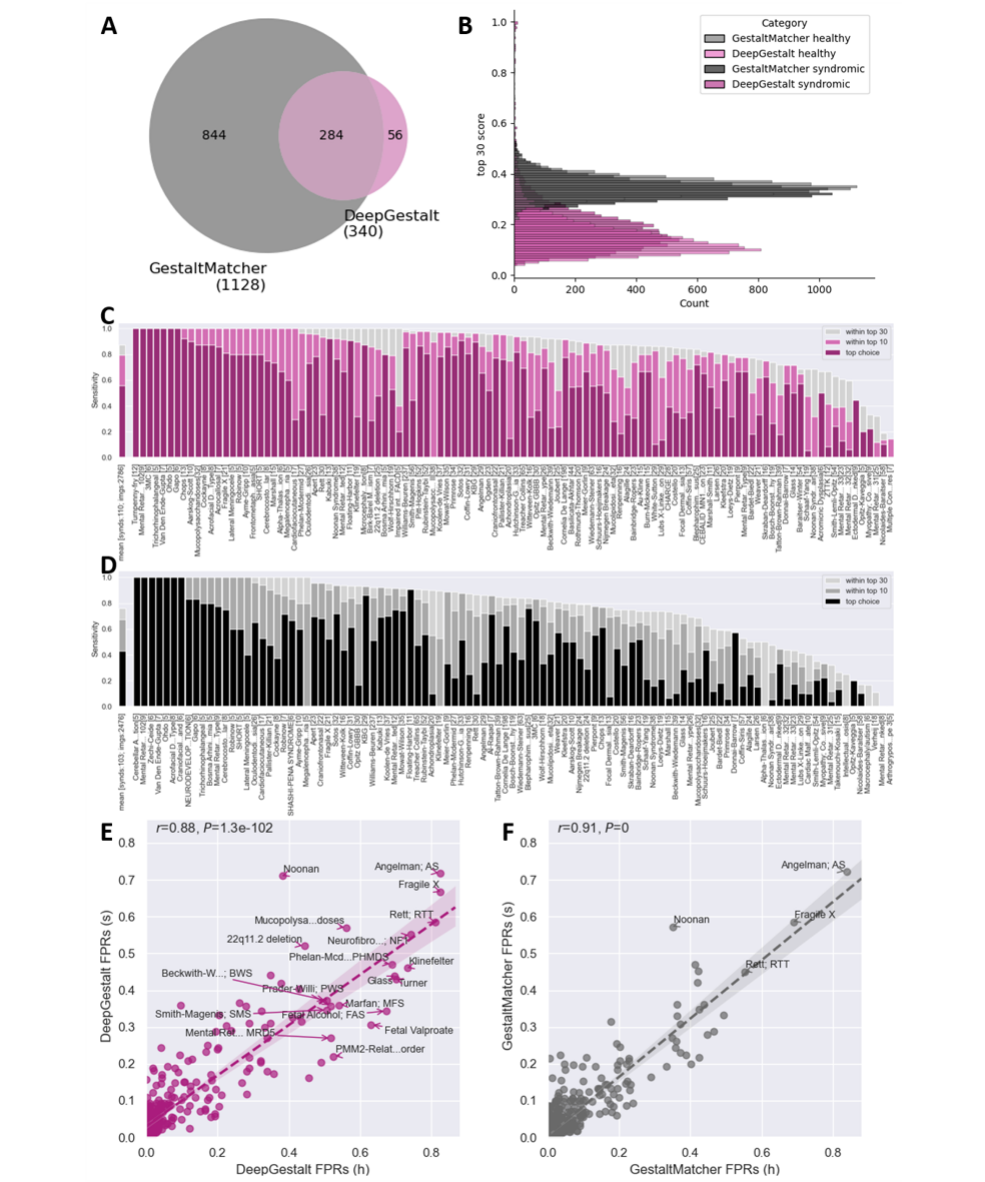
Comparison of DeepGestalt and GestaltMatcher: (A) Venn diagram showing the number of syndromes supported by DeepGestalt (purple) and GestaltMatcher (grey). (B) Histogramme of top-30 scores yielded by DeepGestalt and GestaltMatcher in patient and control images. Sensitivities of (C) DeepGestalt and (D) GestaltMatcher for syndromes featuring at least 5 analyzed images among all patients included images with mean sensitivity averaged by syndrome. Linear regressions of false positive rates of (E) DeepGestalt’s and (F) GestaltMatcher’s top-30 results in matched control (y-axis) and patient (x-axis) images of the literature data set. (High resolution image available in Multimedia Appendix 2). FPR: false positive rate; h: healthy control; s: patient with syndrome.

In total, 4796 images were used to test DeepGestalt, GestaltMatcher, and D-score. To investigate and compare the scope of diagnoses for which DeepGestalt and GestaltMatcher can be used, we counted the number of syndromes suggested by each tool. Among all matches, DeepGestalt and GestaltMatcher suggested a total of 340 and 1128 different syndromes, respectively. There was an intersection of 284 syndromes proposed by both tools (Figure 2A). A total of 56 syndromes were returned only by DeepGestalt, whereas 844 syndromes were exclusively suggested by GestaltMatcher (Figure 2A).

The level of a yielded score is designed to increase with the likelihood of a correct syndrome suggestion. However, this might falsely be interpreted by a user as a direct metric for the probability of a suggested syndrome to be a true positive result. To elucidate the actual distributions, we analyzed the scores returned by DeepGestalt and GestaltMatcher in the top-30 (Figure 2B). Gestalt scores assigned by DeepGestalt ranged predominantly from 0.05 to 0.5, while those assigned by GestaltMatcher were mostly between 0.2 and 0.5. We found that DeepGestalt returned higher scores for syndromic subjects with a peak around 0.16 compared to a peak around 0.1 for the healthy cohort. In contrast, GestaltMatcher scores showed a higher degree of overlap with a peak around 0.33 for the patient cohort and a peak at even higher values around 0.35 for the controls. Overall, the values assigned by GestaltMatcher were higher than those of DeepGestalt but did not differ between patients and controls.

To measure the tools’ quality, we explored their sensitivities for the syndromes included in our data sets. We were able to evaluate 110 syndromes in DeepGestalt and 103 syndromes in GestaltMatcher with at least 5 images per supported syndrome (Figure 2C and D). DeepGestalt and GestaltMatcher both ranked 7 syndromes correctly on top-1 in all cases. The syndromes that were always suggested correctly on rank 1 by both tools were Mental Retardation X-Linked 102, Van Den Ende-Gupta syndrome, and Ohdo syndrome. GestaltMatcher did not suggest the correct diagnosis in the top-30 for 2 syndromes. Overall, DeepGestalt showed higher sensitivities than GestaltMatcher, achieving an average top-1 sensitivity of 56% (SD 29%), an average top-10 sensitivity of 79% (SD 23%), and an average top-30 sensitivity of 88% (SD 18%), while the correct diagnosis was returned by GestaltMatcher at rank 1 on average in 43% (SD 30%), within the top-10 in 67% (29%), and within the top-30 in 76% (SD 26%) of patient images. To directly compare results yielded by the current version with those yielded by an earlier version of DeepGestalt, we also explored the current sensitivities of both tools exclusively on the patient cohort of the “literature image” set. Also on these images, DeepGestalt outperformed GestaltMatcher (top-1 sensitivity 58%, SD 28% vs 38%, SD 24%; top-10 sensitivity 91%, SD 10% vs 79%, SD 19%; and top-30 sensitivity 97%, SD 6% vs 93%, SD 9%; Multimedia Appendix 3).

To test the specificity of syndrome suggestions, we explored false syndrome suggestions by DeepGestalt and GestaltMatcher. The vast majority of syndromes were infrequently suggested by DeepGestalt with only 19 syndromes being suggested among the top-30 ranks in more than 50% of patient or control images (Figure 2E). False syndrome suggestion rates strongly correlated between patient and control images (*r*=0.88). The most frequently falsely suggested syndrome in either cohort was Angelman syndrome (healthy controls false positive rate [FPR]=0.83 and patient cohorts FPR=0.72). False positive rates of GestaltMatcher were lower with only 4 syndromes being suggested among the top-30 ranks in more than 50% of either patient or healthy control images (Figure 2F). GestaltMatcher false positive rates also showed a strong correlation between patient and control images (*r*=0.91) and suggested Angelman syndrome as the most frequent false positive result (healthy controls FPR=0.84 and patient cohorts FPR=0.72). False positive suggestion rates of DeepGestalt and GestaltMatcher strongly correlated in both the controls (*r*=0.91) and the patient cohort (*r*=0.91, Multimedia Appendix 4). Angelman syndrome was not only the syndrome that was most frequently suggested by either tool among the top-30 ranks, but together with Fragile X syndrome, it was also the most frequently implied among the top-10, top-5, and on the first rank (Multimedia Appendix 5).

### Discriminatory Power of GestaltMatcher, DeepGestalt, and D-Score

**Figure 3:**
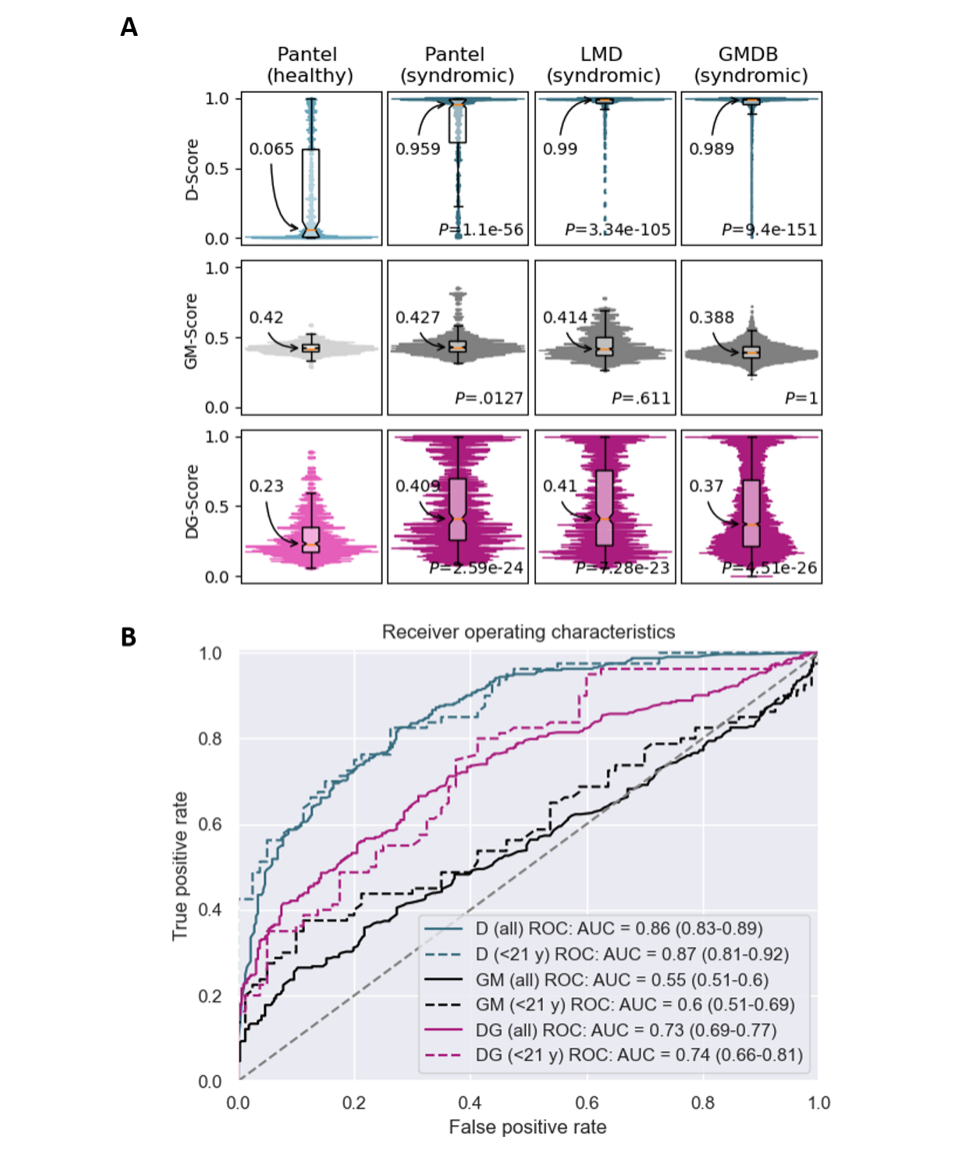
Accuracy of DeepGestalt, GestaltMatcher and D-Score. (A) Distributions of the scores yielded by D-Score (D, turquoise), GestaltMatcher (GM, grey), and DeepGestalt (DG, purple), in the different test sets. (B) Receiver operating characteristic curves (ROC) of D-Score (D, turquoise), GestaltMatcher (GM, grey), and DeepGestalt (DG, purple). (High resolution version available in Multimedia Appendix 6). AUC: area under the curve; GMDB: GestaltMatcher Database; LMD: London Medical Database; ROC: receiver operating characteristic.

To benchmark their ability to discern patients from controls, we examined and compared scores yielded by D-Score and first-rank scores of DeepGestalt and GestaltMatcher (Figure 3A). First-rank DeepGestalt scores were lower in the healthy control cohort (median 0.23, IQR 0.169-0.347) than in the patient cohorts (Pantel syndromic median 0.409, IQR 0.255-0.696; LMD median 0.41, IQR 0.217-0.754; and GMDB median 0.37, IQR 0.21-0.685). First-rank DeepGestalt scores showed high variances and overlapped between patient and control images with lower quartiles of the patient cohorts being between the median and upper quartile of the control cohort.

Median first-rank GestaltMatcher scores were higher than median first-rank DeepGestalt scores but similar between control and patient images (Pantel control GestaltMatcher median 0.42, Pantel syndromic GestaltMatcher median 0.427, LMD GestaltMatcher median 0.414, and GMDB GestaltMatcher median 0.388). First-rank GestaltMatcher scores were less variable than the first-rank DeepGestalt scores.

D-Score distributions differed between the control cohort (median 0.065) and the patient cohorts (Pantel syndromic median 0.959, LMD median 0.99, and GMDB median 0.989) with nonoverlapping IQR. D-Score showed little variability with control scores clustering toward 0 and patient scores toward 1.

As a major metric of the power to discern patient and control images, we investigated the receiver operating characteristic (ROCs) curves of DeepGestalt, GestaltMatcher, and D-Score (Figure 3B). D-Score yielded the highest curve with an area under the curve (AUC) of 0.86. DeepGestalt achieved the second-best outcome with an AUC of 0.73. As expected, GestaltMatcher did not achieve separability of the 2 classes with an AUC of 0.55.

### Confounders of D-Score

To test for performance differences resulting from patient-specific characteristics known to possibly confound face classification systems, we also examined performance metrics on respective subsets (age, sex, ethnicity, and diagnosis) of our cohort.

Distributions of first-rank scores of DeepGestalt and GestaltMatcher were comparable for all age groups regarding both patient and control images (Multimedia Appendix 7). However, D-scores tended to increase with growing age, particularly for control images (Multimedia Appendix 7). Nevertheless, the ROC of D-scores of minors (younger than 21 years of age, AUC=0.87) was not markedly different from the ROC for all age groups (Figure 3B). Upon evaluation of the 17 syndromes included in the Pantel syndromic cohort, first-rank GestaltMatcher scores did not vary depending on a patient’s diagnosis, while we found minor differences in the first-rank DeepGestalt score distributions and strong differences in D-Score distributions (Multimedia Appendix 8).

First-rank DeepGestalt and GestaltMatcher scores did not differ between female and male individuals, whereas D-scores tended to be higher in photos of male individuals than in images of female individuals for both the Pantel syndromic and control cohort. This strong difference was not seen in the larger GMDB cohort (Multimedia Appendix 9).

Strongly significant differences in score distributions of White and other individuals were only seen in the GMDB patient cohort (Multimedia Appendix 10).

## Discussion

### Principal Findings

We evaluated the recently developed algorithms GestaltMatcher and D-Score in comparison to the established DeepGestalt. D-Score provides an estimate for the degree of dysmorphism of a face, while DeepGestalt showed the best sensitivity by rank. GestaltMatcher offers a large number of detectable syndromes.

Capable of achieving the same task of suggesting dysmorphic syndromes based on patient images, DeepGestalt and GestaltMatcher have specific strengths and weaknesses. When comparing the ranges of scores yielded by DeepGestalt and by GestaltMatcher, we found that GestaltMatcher scores on average exceeded DeepGestalt scores. This information is crucial for use in the clinical field, as physicians must be aware that scores obtained through analysis by the new GestaltMatcher cannot be interpreted identically to scores produced by DeepGestalt. While DeepGestalt shows higher sensitivities for the syndromes it supports, the number of syndromes supported by GestaltMatcher is more than 3 times greater. The average sensitivities of both tools are sufficient for diagnostic decision support. The sensitivity of DeepGestalt version 21.5.0 tested here is comparable to that of version 19.1.7 tested in a previous study [20], suggesting that the version updates carried out so far have had a minor impact on DeepGestalt’s performance. Nevertheless, users need to be aware that specificities for a few officially supported syndromes are very low, for example, GestaltMatcher failed to identify 2 syndromes (Mental Retardation X-linked and Arthrogryposis, distal type 3). Less syndromes are frequently falsely suggested by GestaltMatcher than by DeepGestalt, implying GestaltMatcher has a greater specificity. This could be expected as GestaltMatcher includes a much larger number of syndromes than DeepGestalt, lowering the chance of individual syndromes appearing as a match. The tendency of the 2 algorithms to falsely suggest certain syndromes correlated strongly. Among the most frequent false suggestions were Angelman syndrome, Fragile X syndrome, and Rett syndrome which feature rather mild facial dysmorphism. Thus, caution is needed when interpreting classification results listing such diagnoses.

First-rank GestaltMatcher scores of affected and unaffected cases overlapped largely, while surprisingly, unaffected cases received a higher median score than affected cases. Thus, GestaltMatcher can currently not be used to screen for individuals with dysmorphic syndromes. In our experiment, the scores assigned by DeepGestalt allowed for a certain separability between affected and unaffected cohorts. Nevertheless, this accuracy does not enable the identification of patients who are dysmorphic with great certainty. The best accuracy to differentiate between affected and unaffected was found for D-Score. Limiting the test set to the age group predominantly seen for syndrome evaluation (under 21 years of age) slightly improved the accuracy.

Notably, in the patient cohort of Pantel et al [20], D-Score yielded the highest scores in syndromes with a strong facial dysmorphism (highest median score in Apert syndrome) and lower scores in syndromes with mild facial features (lowest median score in Rett syndrome). D-Scores of control images increased with age, suggesting it is predominantly trained on children. We suggest that clinicians may exercise caution when interpreting scores obtained for patients older than 21 years of age, as older faces may automatically be recognized as dysmorphic due to signs of aging. D-Score assigned higher scores to images of healthy male individuals than healthy female individuals, which needs to be taken into consideration when interpreting the obtained score values. Ethnic background was not shown to influence the performance of either of the 3 algorithms tested. However, future studies ideally include ethnically more diverse test sets.

### Strengths and Limitations

To our knowledge, we performed the most extensive evaluation of DeepGestalt thus far and the first comprehensive evaluations of GestaltMatcher and D-Score. Nevertheless, there are limitations to the analyses we conducted in this study. First, we restricted our analysis of GestaltMatcher to the first 30 results to match the number of results of DeepGestalt provided via Face2Gene CLINIC. As GestaltMatcher can match a far larger number of patients and syndromes, an analysis including all possible 100 results, using a larger number of syndromes and even images of undiagnosed patients, would provide a more comprehensive assessment of its performance. Second, the number of healthy control images is limited, and we tested for possible confounders of D-Score, which constrained the number of (both patient and control) cases in some subgroups. To evaluate the influence of these and other possible confounders such as facial hair, glasses, positioning, and lighting, further research using larger test sets is necessary. Third, a direct comparison of the D-score to other algorithms, designed to discern images of patients who are dysmorphic from those of healthy controls is needed. Recently, Porras et al [13] described such a tool achieving an AUC of 0.94 on a private data set. Unfortunately, no tool other than D-score is available for the broader public for testing purposes. Thus, benchmarking different tools on the same data set has not been possible.

### Future Directions

Even though the algorithms cannot replace the expertise of a physician in medical genetics, they could potentially play a complementary role in future decision-making. D-Score showed the best separation between syndromic and nonsyndromic images, giving it a potential future role as a screening tool. GestaltMatcher could be applied as a “second-line” test to analyze patients’ images that yielded no or only low-similarity matches in the DeepGestalt analysis.

Physicians must exercise caution when using tools tested in this study, as the scores assigned vary between the tools and can thus not be interpreted identically. A prospective study, as recently conducted to assess the application of DeepGestalt in the clinical field [21], is needed to further investigate the functionality of GestaltMatcher and D-Score in a point-of-care setting.

## Appendix

Multimedia Appendix 1 Code and data required to reproduce statistical results from this study.

Multimedia Appendix 2 Comparison of DeepGestalt and GestaltMatcher (high resolution).

Multimedia Appendix 3 Sensitivities of (A) DeepGestalt and (B) GestaltMatcher in the patient cohort of the "literature image" set. Higher bars indicate higher mean sensitivity, averaged by syndrome. Far left: mean sensitivity across all represented syndromes.

Multimedia Appendix 4 Linear regression of DeepGestalt’s (x-axis) and GestaltMatcher’s (y-axis) false positive rates (FPRs) in matched control (A) and patient (B) images of the “literature image” set.

Multimedia Appendix 5 Linear regressions of false positive rates (FPRs) of DeepGestalt’s (purple) and GestaltMatcher’s (grey) in patient (x-axis) matched control (y-axis) images of the literature data set. (A,B) Calculated from top-10 results; (C,D) Calculated from top-5 results; (E,F) Calculated from top-1 suggestions only.

Multimedia Appendix 6 Accuracy of DeepGestalt, GestaltMatcher and D-Score (high resolution).

Multimedia Appendix 7 Distributions of the scores yielded by D-Score (turquoise), GestaltMatcher (grey), and DeepGestalt (purple) in the different test sets, separated by age groups. h: healthy control, s: patient with syndrome, P: Pantel image set, G: GestaltMatcher Database.

Multimedia Appendix 8 Distributions of the scores yielded by D-Score (turquoise), GestaltMatcher (grey), and DeepGestalt (purple) in the Pantel patient set separated by diagnosis. The *P* values refer to differences to matched healthy controls.

Multimedia Appendix 9 Distributions of the scores yielded by D-Score (turquoise), GestaltMatcher (grey), and DeepGestalt (purple) separated by sex. Heal: healthy control, synd: patients with a syndrome, f: female, m: male, GMDB: GestaltMatcher Database.

Multimedia Appendix 10 Distributions of the scores yielded by D-Score (turquoise), GestaltMatcher (grey), and DeepGestalt (purple) separated by ethnicity. Heal: healthy control, synd: patients with a syndrome, Wh: White, Oth.: Other, GMDB: GestaltMatcher Database.

## Abbreviations

AUC: area under the curve
AUROC: area under the receiver operating characteristic
FPR: false positive rate
GMDB: Gestalt Matcher Database
LMD: London Medical Database
NGP: next-generation phenotyping
ROC: receiver operating characteristic

## Data Availability

Characteristics of the literature image data analyzed during this study are available in a previous publication [20]. Image data from the London Medical Database analyzed during this study are available in the Face2Gene Library [31]. Image Data of the GestaltMatcher Database analyzed during this study are available therein [32]. All data generated during this study are included in this published article and Multimedia Appendices 1-10. High resolution versions of Figures 2 and 3 can be found in Multimedia Appendices 2 and 6.
