# Supplementary figures and images for: Validation of 3 Computer-Aided Facial Phenotyping Tools (DeepGestalt, GestaltMatcher, and D-Score): Comparative Diagnostic Accuracy Study
Source: J Med Internet Res. 2024 Mar 13;26:e42904. doi: 10.2196/42904 (PMC10973953; doi:10.2196/42904)

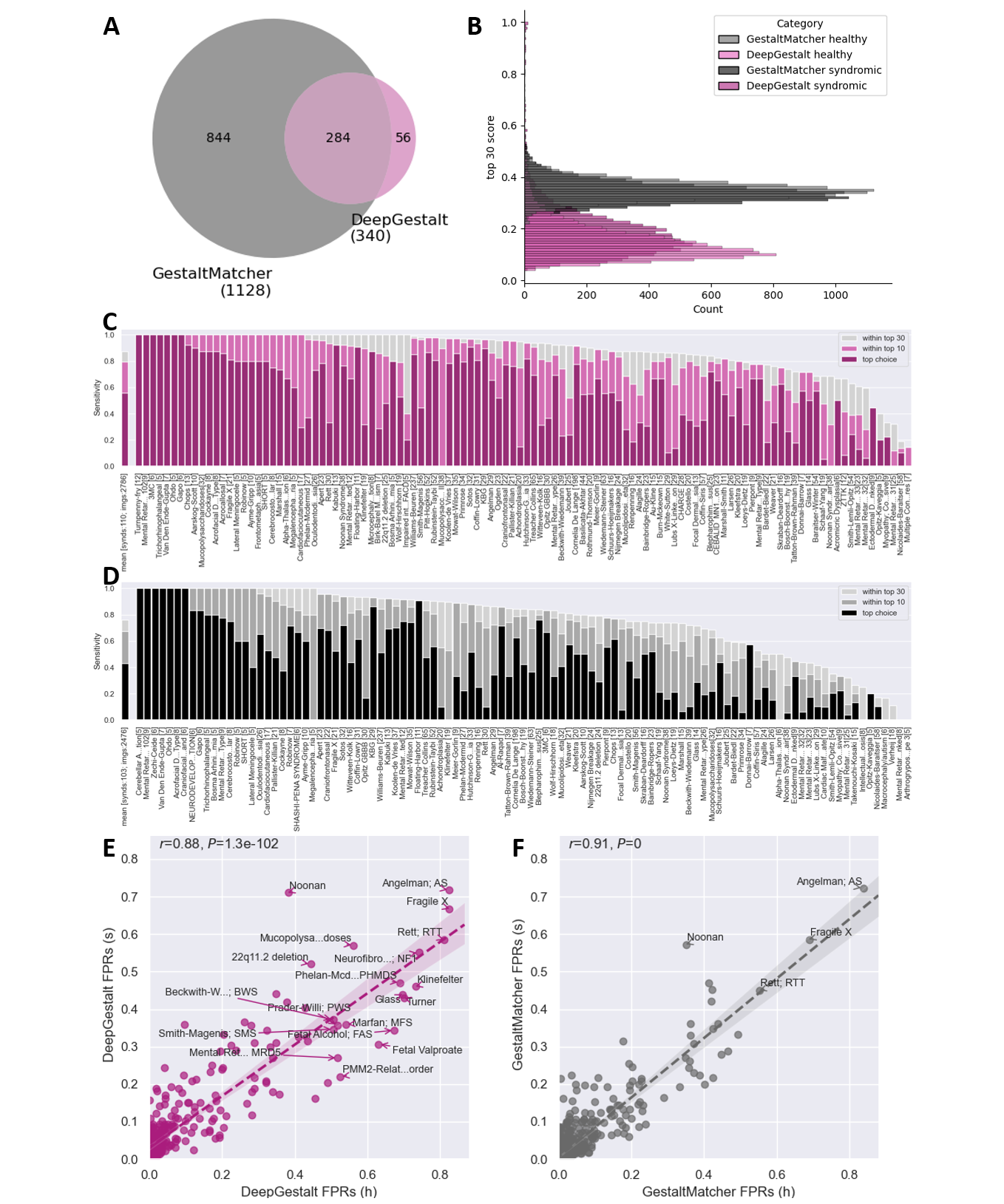

Supplement: Multimedia Appendix 2 [file jmir_v26i1e42904_app2.png]

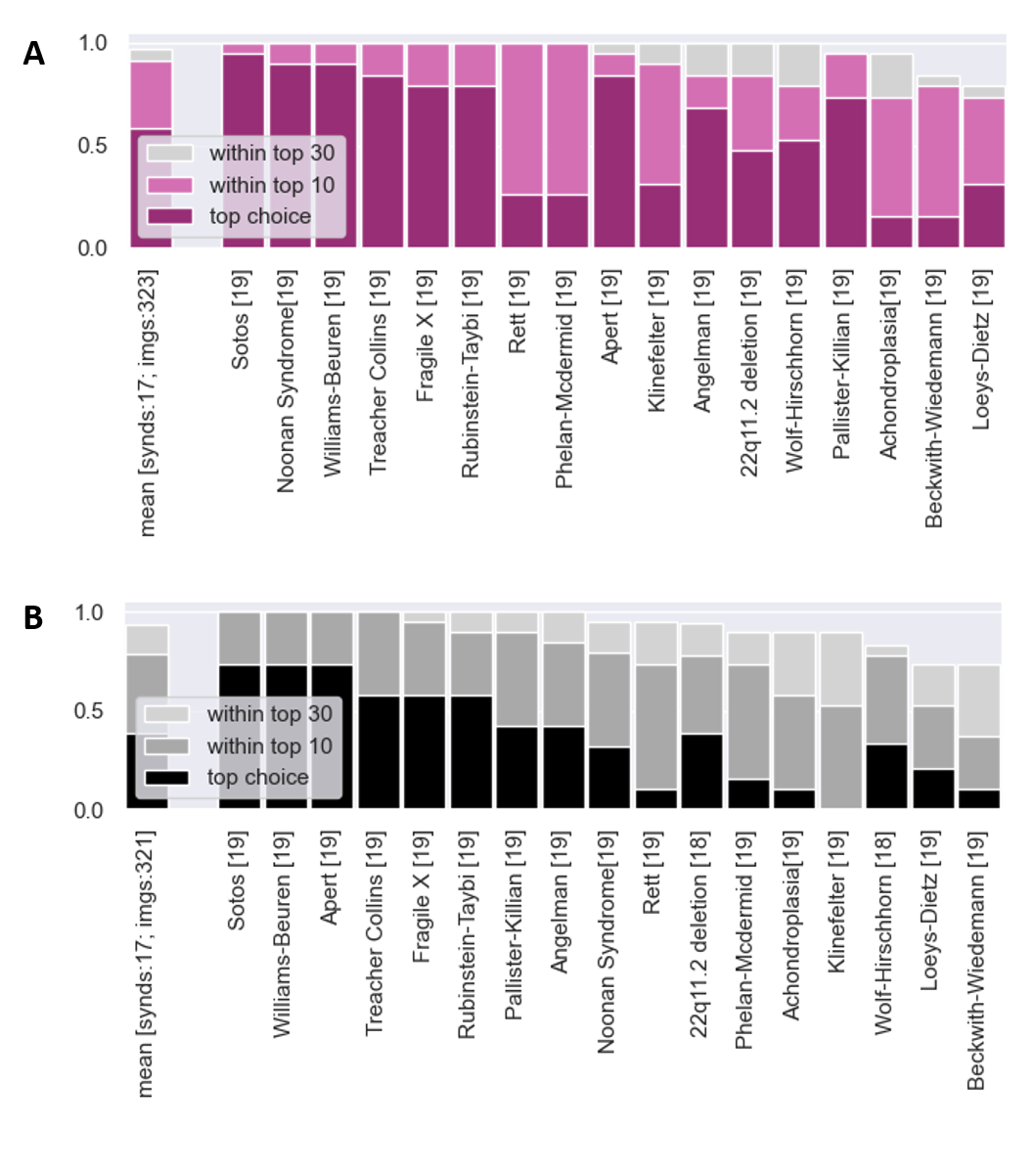

Supplement: Multimedia Appendix 3 [file jmir_v26i1e42904_app3.png]

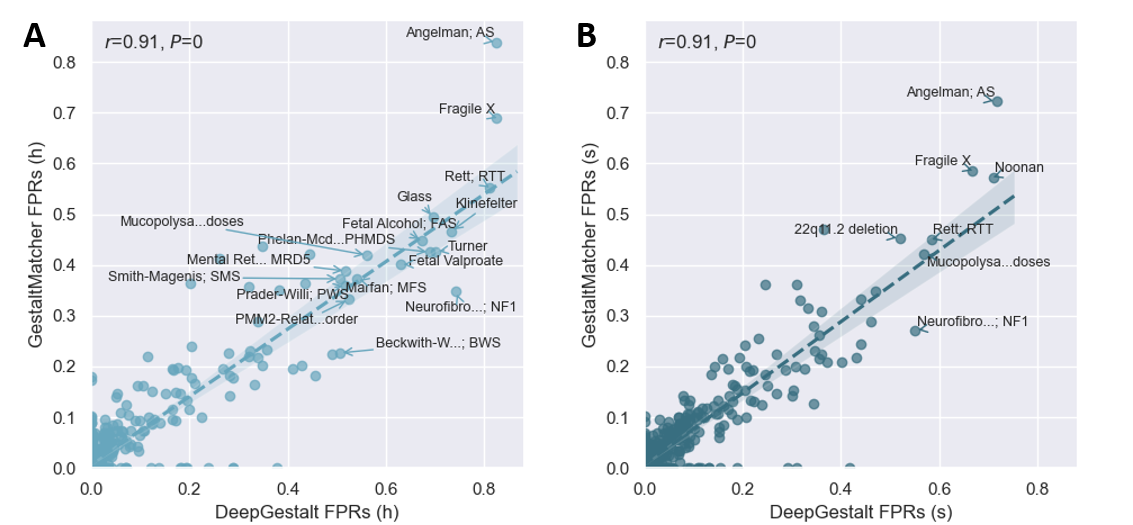

Supplement: Multimedia Appendix 4 [file jmir_v26i1e42904_app4.png]

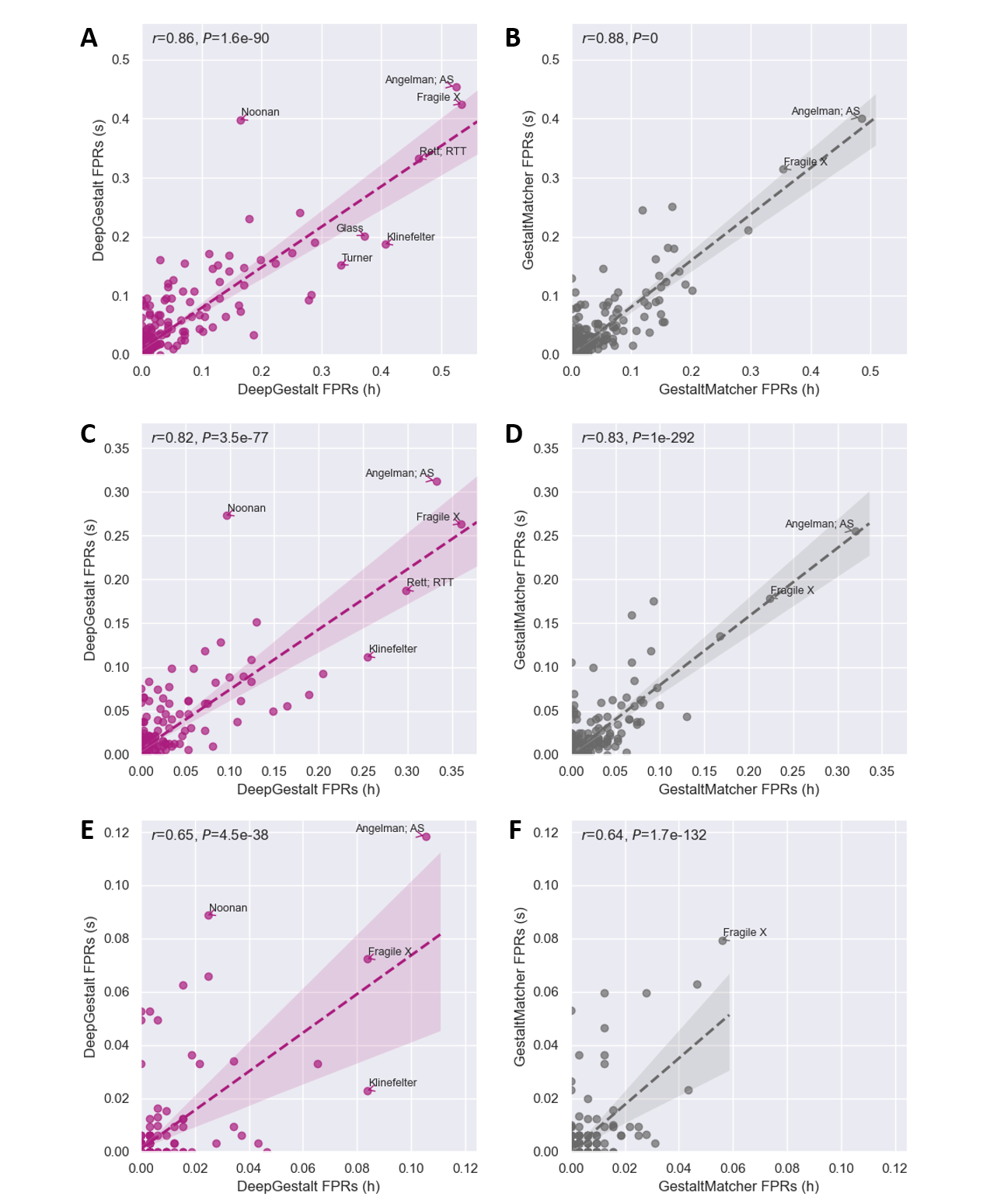

Supplement: Multimedia Appendix 5 [file jmir_v26i1e42904_app5.png]

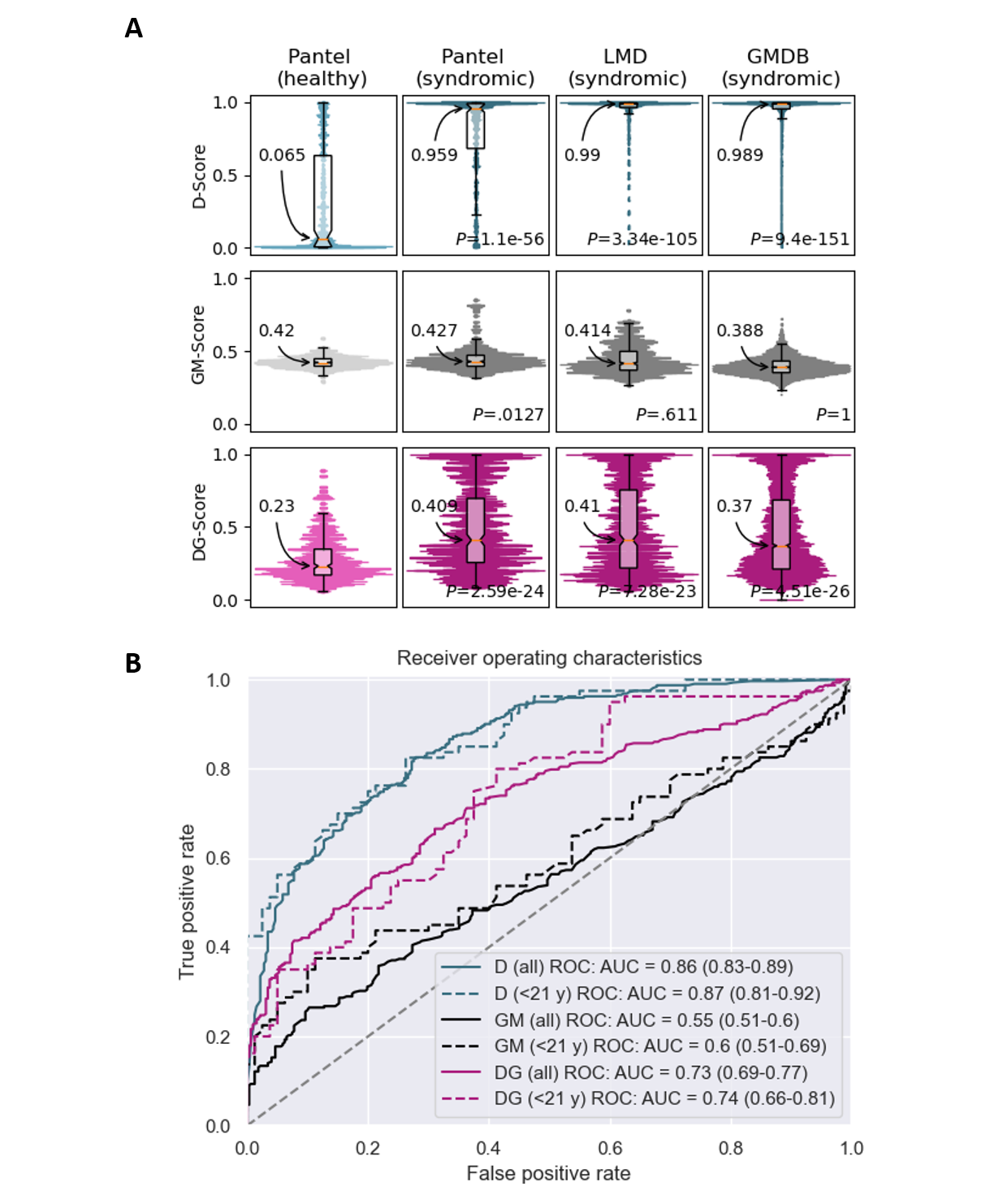

Supplement: Multimedia Appendix 6 [file jmir_v26i1e42904_app6.png]

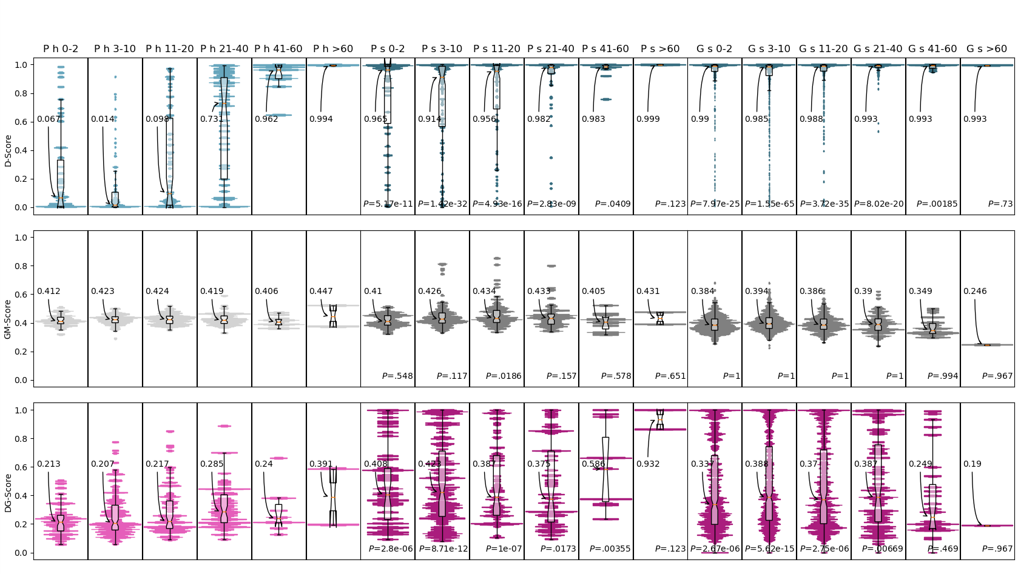

Supplement: Multimedia Appendix 7 [file jmir_v26i1e42904_app7.png]

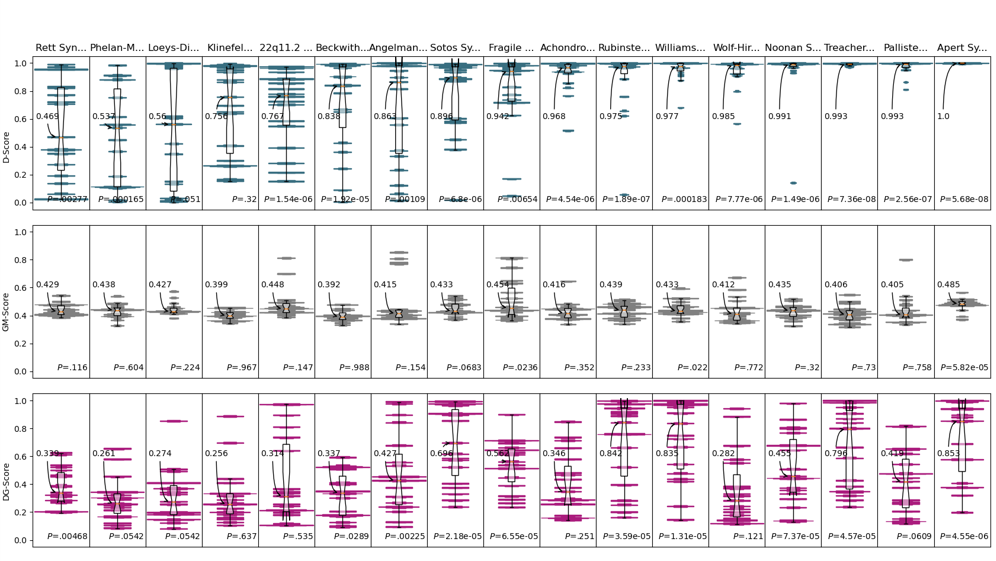

Supplement: Multimedia Appendix 8 [file jmir_v26i1e42904_app8.png]

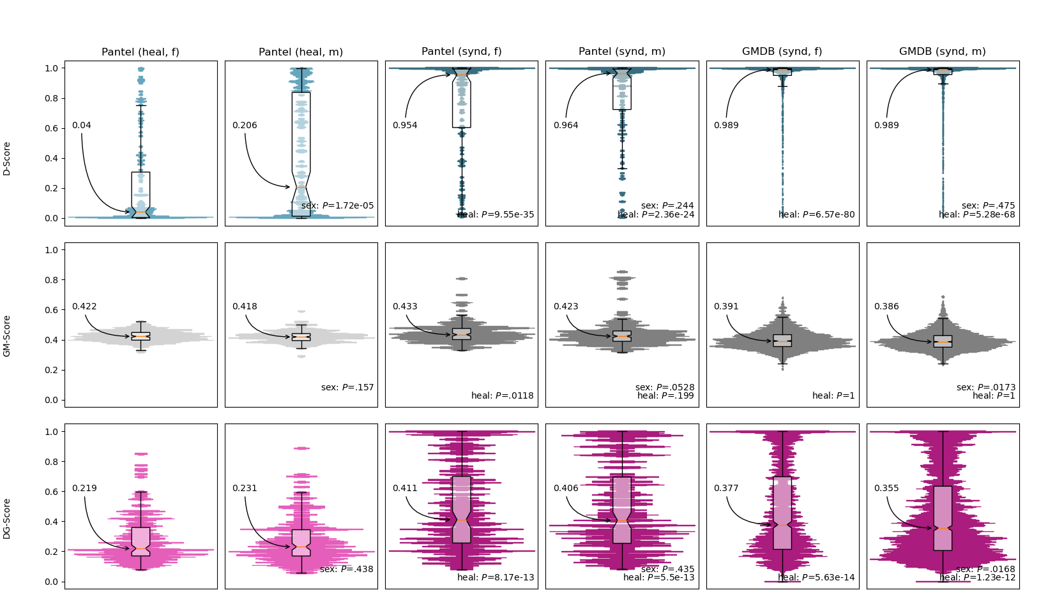

Supplement: Multimedia Appendix 9 [file jmir_v26i1e42904_app9.png]

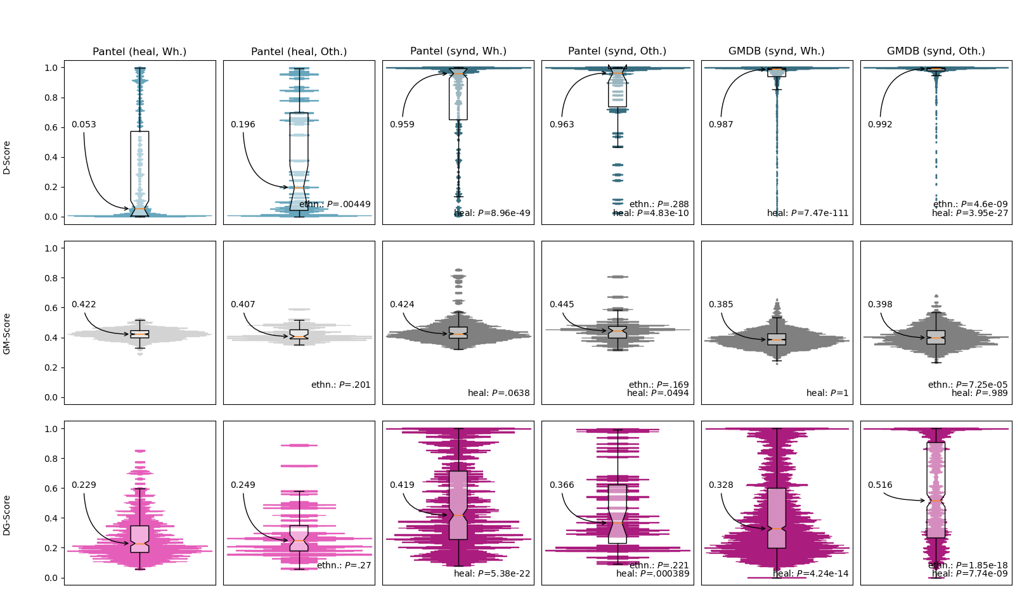

Supplement: Multimedia Appendix 10 [file jmir_v26i1e42904_app10.png]
